# D‐dopachrome tautomerase in adipose tissue inflammation and wound repair

**DOI:** 10.1111/jcmm.12936

**Published:** 2016-09-07

**Authors:** Bong‐Sung Kim, Pathricia V. Tilstam, Soo Seok Hwang, David Simons, Wibke Schulte, Lin Leng, Maor Sauler, Bergita Ganse, Luisa Averdunk, Rüdger Kopp, Christian Stoppe, Jürgen Bernhagen, Norbert Pallua, Richard Bucala

**Affiliations:** ^1^ Department of Plastic and Reconstructive SurgeryHand Surgery ‐ Burn CenterRWTH Aachen UniversityAachenGermany; ^2^ Section of RheumatologyDepartment of MedicineYale University School of MedicineNew HavenCTUSA; ^3^ Department of ImmunologyYale University School of MedicineNew HavenCTUSA; ^4^ German Cancer Research Center (DKFZ)HeidelbergGermany; ^5^ Section of Pulmonary, Critical Care, and Sleep MedicineDepartment of MedicineYale University School of MedicineNew HavenCTUSA; ^6^ Department of Orthopedic Trauma SurgeryRWTH Aachen UniversityAachenGermany; ^7^ Department of Intensive Care MedicineRWTH Aachen UniversityAachenGermany; ^8^ Chair of Vascular BiologyInstitute for Stroke and Dementia ResearchKlinikum der Universität MünchenLudwig‐Maximilians‐UniversityMunichGermany; ^9^ Munich Cluster for Systems Neurology (SyNergy)MunichGermany

**Keywords:** D‐dopachrome tautomerase, D‐DT, MIF, adipose tissue, inflammation, wound

## Abstract

D‐dopachrome tautomerase (D‐DT/MIF‐2) is a member of the macrophage migration inhibitory factor (MIF) cytokine superfamily, and a close structural homolog of MIF. MIF and D‐DT have been reported to be involved in obesity, but there is little known about the regulation of D‐DT in adipose tissue inflammation and wound healing. Subcutaneous adipose tissue was collected from 54 healthy donors and 28 donors with acutely inflamed wounds undergoing wound debridement. In addition, epididymal fat pads of mice were injected with lipopolysaccharide to study receptor expression and cell migration *in vivo*. D‐DT protein levels and mRNA expression were significantly decreased in subcutaneous adipose tissue adjacent to acutely inflamed wounds. D‐DT improved fibroblast viability and increased *proliferation in vitro*. While D‐DT alone did not have a significant effect on *in vitro* fibroblast wound healing, simultaneous addition of neutralizing MIF antibody resulted in a significant improvement of fibroblast wound healing. Interestingly, expression of the MIF and D‐DT receptor *CD74* was down‐regulated while the MIF receptors *CXCR2* and *CXCR4* were up‐regulated primarily on macrophages indicating that the MIF‐CXCR2/4 axis may promote recruitment of inflammatory cells into adipose tissue. Our results describe a reciprocal role of D‐DT to MIF in inflamed adipose tissue, and indicate that D‐DT may be beneficial in wound repair by improving fibroblast survival and proliferation.

## Introduction

Wound repair is a complex response by the host to tissue damage, and wound healing disorders contribute to significant morbidity and excess health care expenditures 1. Acute wound healing disorders occur at areas of adipose tissue inflammation 2, 3, which can lead to life‐threatening complications and extended soft tissue defects requiring complex reconstruction procedures 4. In the past several years, adipose tissue has been determined to be an important endocrine organ that orchestrates diverse responses by the release of specialized cytokines in an autocrine and paracrine manner 5. Subcutaneous adipose tissue contributes to cutaneous wound healing through the secretion of adipokines and the differentiation of progenitor cells into keratinocytes and fibroblasts 6, 7, 8, 9, 10. Obesity has been implicated in a number of autoimmune diseases and the increasing number of obese patients with impaired wound healing has generated significant interest in the function of adipokines during wound repair 9. One adipokine that has been recently characterized in obesity and insulin resistance is D‐dopachrome tautomerase (D‐DT) 11. D‐DT was first described by Odh and colleagues as an enzyme in human melanoma, human liver and several rat organs which tautomerizes D‐dopachrome, but its precise role in body physiology remained unknown for many years 12. The description of structural and functional similarities between D‐DT and the innate cytokine macrophage migration inhibitory factor (MIF) recently advanced our understanding of D‐DT significantly 13, 14 and led to the alternative designation of D‐DT as MIF‐2 15.

We previously reported increased MIF levels in subcutaneous adipose tissue in participants with acute wound healing disorders and proposed that MIF may mediate the recruitment of inflammatory cells 4. While MIF and D‐DT share functional similarities, expression data suggest that during chronic adipose tissue inflammation and obesity, the two cytokines have opposing or complementary roles 11. Migration inhibitory factor promotes chronic adipose tissue inflammation and its expression positively correlates with obesity and insulin resistance, whereas D‐DT expression shows a negative correlation 11, 16. The goal of this study was to elucidate more precisely the function of D‐DT in subcutaneous adipose tissue adjacent to acute wound healing disorders.

## Materials and methods

### Adipose tissue samples

Healthy adipose tissue (HAT) was collected from healthy patients undergoing elective plastic surgery, whereas inflammatory adipose tissue (IAT) was harvested from acute wound healing disorders. Those were defined as wounds caused by external trauma, iatrogenic manipulation and surgical intervention, which were not resolved within a period of 4 weeks after trauma 2, 3. All cases showed classic signs of local inflammation (*tumour*,* calor*,* rubor*,* dolour* and *functio laesa*) and had negative bacterial swab samples at the time of tissue harvest. The adipose tissue was excised and sample preparation was carried out as previously described 4. All surgeries were performed in the Department of Plastic Surgery, Hand Surgery – Burn Center of the RWTH University Hospital Aachen, Germany. The study was approved by the ethics committee of the RWTH Aachen University (EK 163/07), with written informed consent provided by patients or relatives. All experiments were performed in compliance with the Declaration of Helsinki Principles.

### Tissue homogenization and detection of total protein

Whole adipose tissue samples were homogenized as described earlier 17. Total protein content of whole adipose tissue samples was measured by the DC Protein Assay (Bio‐Rad Laboratories GmBH, Munich, Germany) according to the manufacturer's instructions.

### Human and murine D‐DT Enzyme linked immunosorbent assay (ELISA)

D‐dopachrome tautomerase protein levels of human adipose tissue homogenates and murine epididymal fat pads were measured by ELISA as previously reported 13.

### Real‐time PCR

Messenger RNA from human adipose tissue and murine epididymal fat pads was extracted by the RNeasy Mini Kit (Qiagen NV, Venlo, The Netherlands) and cDNA synthesis was performed with the QuantiTect Reverse Transcription Kit (Qiagen Inc., Valencia, CA, USA) following the manufacturer's instructions. Complementary DNA was subject to quantitative real‐time (RT) PCR using the SYBR^®^ Green Master Mix (Bio‐Rad Laboratories, Inc., Hercules, CA, USA). Primers used in this study are listed in Table S1. For human samples, *GAPDH* was used as an internal control, while in mice *β‐actin* served as a reference. Data were analysed with the comparative cycle time (CT) method.

### Immunohistochemical staining for D‐DT, CXCR2, CXCR4 and CD74

Paraffin embedded whole adipose tissue samples were sectioned and stained with haematoxylin/eosin and 3,3′‐Diaminobenzidine (Invitrogen, Thermo Fisher Scientific, MA, USA). Polyclonal rabbit anti‐human D‐DT antibody 13, CD74 (Invitrogen, Thermo Fisher Scientific, Waltham, Massachusetts, United States), anti‐human CXCR2 and CXCR4 (both Abcam, Cambridge, UK) were used for immunohistochemical staining. Istoype controls served as negative control, testis and bone marrow served as positive controls.

### CXCR2, CXCR4 and CD74 staining by flow cytometry

Adipocytes and adipose tissue macrophages (ATM) were isolated from lipopolysaccharide (LPS) or PBS‐injected mice for flow cytometry by collagenase digestion as previously described 18, 19. Used antibodies are found in Table S2. ATMs were stained for CD45, CD11b and F4/80. Adipocytes were stained for CR4. Fluorescence minus one controls were included for both cell types. Samples were measured on a LSR flow cytometer (BD Bioscience, San Jose, CA, USA) and the data were analysed using FlowJo (FlowJo LLC, Ashland, OR, USA).

### Isolation of human dermal fibroblasts

Human dermal fibroblasts (HDF) were isolated from healthy patients as described earlier 20 and cultured at 37°C in a humidified atmosphere at 5% CO_2_.

### Measurement of cell viability by the alamarBlue^®^ assay

Viability of HDF was measured by the alamarBlue^®^ assay (Invitrogen, Thermo Fisher Scientific, Darmstadt, Germany) according to the manufacturer's guidelines. Human dermal fibroblasts were seeded in 96 well plates. The following day, alamarBlue^®^ reagent was added to untreated HDF, this time‐point is referred to as the baseline viability. All wells were washed and incubated with supernatants from HAT and IAT, supplemented with varying concentrations of recombinant human D‐DT. After 24 hrs, another alamarBlue^®^ measurement was performed.

### Measurement of cell proliferation by the bromodeoxyuridine ELISA

Proliferation of HDF was analysed by the CytoSelect^™^ bromodeoxyuridine (BrdU) Cell Proliferation Elisa Kit (Cell Biolabs Inc., San Diego, CA, USA). Human dermal fibroblasts were seeded in 96 well plates. The following day, cells incubated with supernatants from HAT and IAT, supplemented with varying concentrations of recombinant human D‐DT for 24 hrs. BrdU incorporation was measured according to the manufacturer's instructions.

### *In vitro* wound healing assay

*In vitro* wound healing was performed by a standard *in vitro* wound healing assay 21. Briefly, HDF monolayers were scratched by a 200 μl pipette tip. Medium containing 10% FCS served as a positive control, while medium containing 0.5% FCS served as a negative control. Human dermal fibroblasts were incubated with supernatants for 16 hrs, which were supplemented with different concentrations of recombinant human D‐DT. 10 μg/ml Mitomycin C (Sigma Aldrich Chemie GmbH, Taufkirchen, Germany) was utilized to examine proliferation and migration‐related wound healing. The MIF antibody III.D.9 was used to show MIF‐dependent effects as reported earlier 22. Photographs were acquired at 0 and 16 hrs after the scratch. The migration (addition of Mitomycin C) and migration/proliferation distance (absence of Mitomycin C) were deduced by comparing the 0 and 16 hrs photographs by the Software Photoshop CS5 (Adobe Systems, Mountain View, CA, USA).

### *In vivo* macrophage migration model

*In vivo* macrophage migration into epididymal fat pads of mice was performed as published earlier 4, 23. Ten‐week‐old male C57BL/6 wild‐type (WT) mice purchased from Charles River Laboratory (Wilmington, DE, USA) were used. All mice were fed with standard chow and water *ad libitum* and experiments were approved by Yale University's Institutional Animal Care and Use Committee (2015‐10756). Five mg/kg LPS (Sigma‐Aldrich, St. Louis, MO, USA) only, LPS together with either 20 μg mouse D‐DT or the equal amount of mouse MIF was injected into the epididymal fat pads *via* a small incision to induce local adipose tissue inflammation. Control mice were treated with the equal volume of PBS. Thioglycollate elicited peritoneal macrophages (PM) were labelled with Cell Tracker Green CMFDA (Cell Tracker Green CMFDA Dye; Life Technologies, Carlsbad, CA, USA). Next, labelled PMs were injected retro‐orbitally. Mice were killed after 48 hrs and epididymal fat tissue was harvested, and the stromal vascular fraction (SVF) was isolated. The isolated ATM were stained with Cd11b AlexaFluor700 (eBioscience, San Diego, CA, USA), F4/80 eFluor450 (eBioscience) and Cd45 APC (Biolegend, San Diego, CA, USA) as reported earlier 4.

### Statistical analysis

For all statistical analysis and diagrams, the software GraphPad Prism^®^ (GraphPad Software, Inc., La Jolla, CA, USA) was used. Data were expressed as mean ± S.E.M. After testing for normal distribution using the Shapiro–Wilk‐W‐test, statistical differences between groups were calculated by one‐way ANOVA in case of three or more variables and *t*‐test in case of two variables. *Post hoc* testing was performed using the Bonferroni‐test. A *P*‐value of <0.05 was considered as significant.

## Results

### Subjects

Healthy adipose tissue samples were obtained from 54 patients [23 male and 31 female, mean age: 46.65 ± 2.16 years, mean body mass index (BMI): 30.03 ± 1.065; Table S3]. Inflammatory adipose tissue was obtained from 28 patients (15 male and 13 female, mean age: 52.75 ± 3.29 years, mean BMI: 30.81 ± 2.634) (Table S4).

### Decreased D‐DT protein levels and mRNA expression in adipose tissue from wound healing disorders

**Figure 1:**
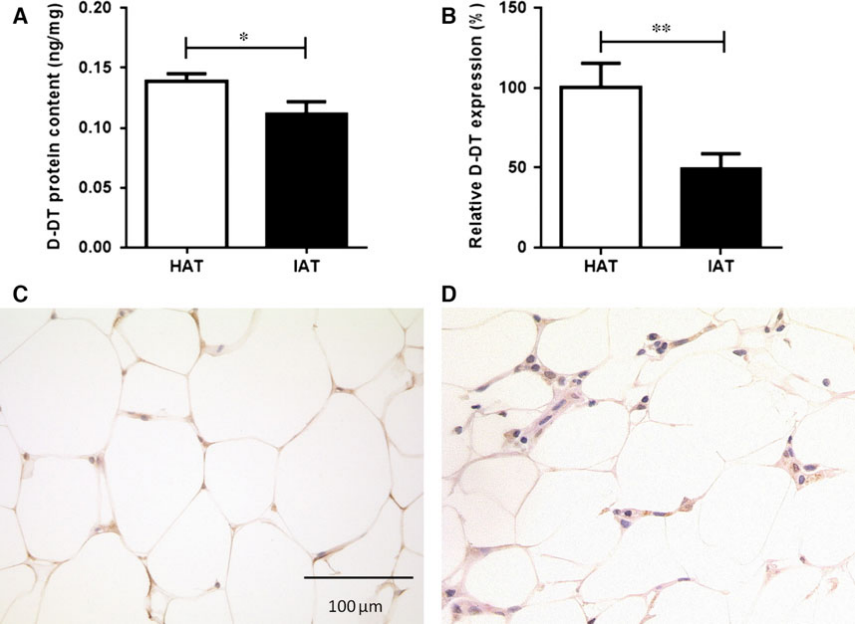
Levels of D‐DT in native HAT and IAT. Adipose tissue from healthy donor sites and acutely inflamed donor sites were homogenized. D‐DT protein levels were analysed by ELISA and D‐DT mRNA levels were measured by quantitative real‐time PCR. (**A**) D‐DT protein levels were significantly decreased in IAT compared to native HAT. Data are presented as mean ± S.E.M., *n* = 54–28, two‐tailed Student's *t*‐test. (**B**) Relative *D‐DT* expression levels in adipose tissue derived from HAT and IAT showed a decrease of *D‐DT* expression in IAT. Data are presented as mean ± S.E.M., *n* = 13–16, two‐tailed Student's *t*‐test. Statistically significant differences are indicated by asterisks (*P < 0.05, **P < 0.01). D‐DT expression was analysed by immunohistochemistry on formalin fixed whole adipose tissue of (**C**) HAT and (**D**) IAT samples (magnification 400 × ). Shown are representative pictures of D‐DT staining.

To study the role of D‐DT in the context of wound healing, D‐DT protein levels from HAT and IAT were measured by ELISA. Inflammatory adipose tissue showed significantly reduced D‐DT levels when compared to HAT (Fig. 1A). Simultaneously, *D‐DT* mRNA expression was analyzed by quantitative real‐time PCR in order to determine if attenuated D‐DT protein levels were related to a down‐regulation of gene expression. Similar to the D‐DT protein levels, mRNA expression of *D‐DT* was decreased in IAT when compared with HAT (Fig. 1B). Immunohistochemical staining also indicated a lower expression of D‐DT protein in mature adipocytes in IAT (Fig. 1C). Positive and negative controls can be found in Figure S1.

### D‐DT levels do not correlate with the donor's body mass index

Given the reported positive correlation of MIF protein levels and the BMI of HAT donor's 4 and elevated MIF plasma levels in obese patients 16, we also examined the correlation of D‐DT levels with the BMI of HAT donor patients and observed that unlike MIF 4, D‐DT did not correlate with the BMI of the donor in HAT (Spearman *r* = 0.1664; Fig. S2).

### D‐DT increases the viability and proliferation of HDF

**Figure 2:**
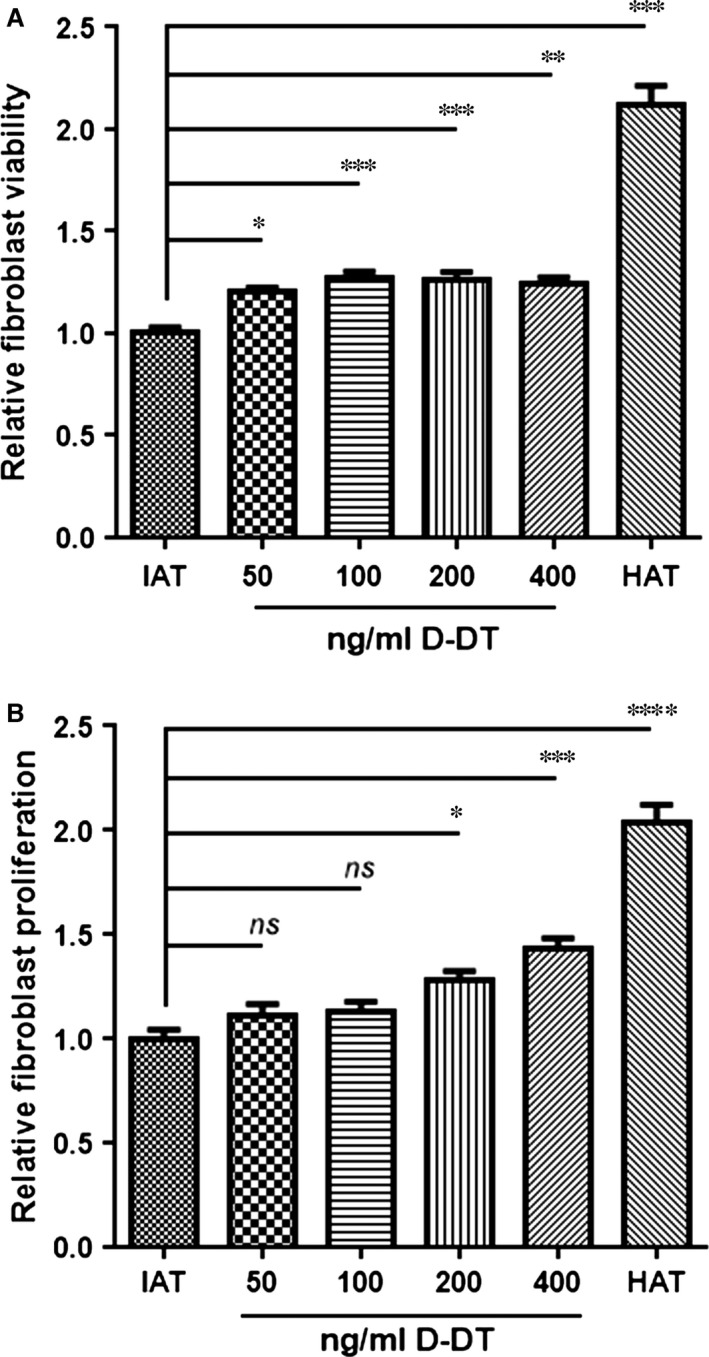
*In vitro* viability and proliferation assay of HDF. Viability and proliferation were assessed for HDF cells. And for both assays, the cells were incubated with supernatants from HAT and IAT supplemented with varying concentrations of recombinant human D‐DT for 24 hrs. (**A**) Viability of HDF was measured by the alamarBlue^®^ assay after 24 hrs stimulation with HAT or IAT supernatants supplemented with D‐DT. Cell viability increased in a dose‐dependent manner, once cells were treated with D‐DT. Graphs represent mean ± S.E.M., n =  14–16 from three independent experiments; one‐way anova with Bonferroni's multiple comparison post‐test. (**B**) HDF proliferation was analysed by the CytoSelect^™^ assay after 24 hrs stimulation with HAT or IAT supernatants with recombinant human D‐DT. D‐DT showed an induction of HDF proliferation in a dose‐dependent manner. Graphs represent mean ± S.E.M., n = 7 from two independent experiments; one‐way anova with Tukey's multiple comparisons test (*P < 0.05, **P < 0.01, ***P < 0.001, ****P < 0.0001).

To evaluate the role of D‐DT in wound healing, we measured the effect of D‐DT on the viability and proliferation of HDFs. Human dermal fibroblasts incubated with supernatants from IAT showed significantly decreased cell viability compared to HDF treated with supernatants from HAT. However, once HDF were cultured with IAT supernatants and treated with D‐DT, the cell viability increased significantly in a dose‐dependent manner (Fig. 2A). We observed that D‐DT also induced HDF proliferation in a dose‐dependent manner (Fig. 2B).

### Increased wound healing in *in vitro* scratch assay of HDF monolayers by D‐DT and anti‐MIF

**Figure 3:**
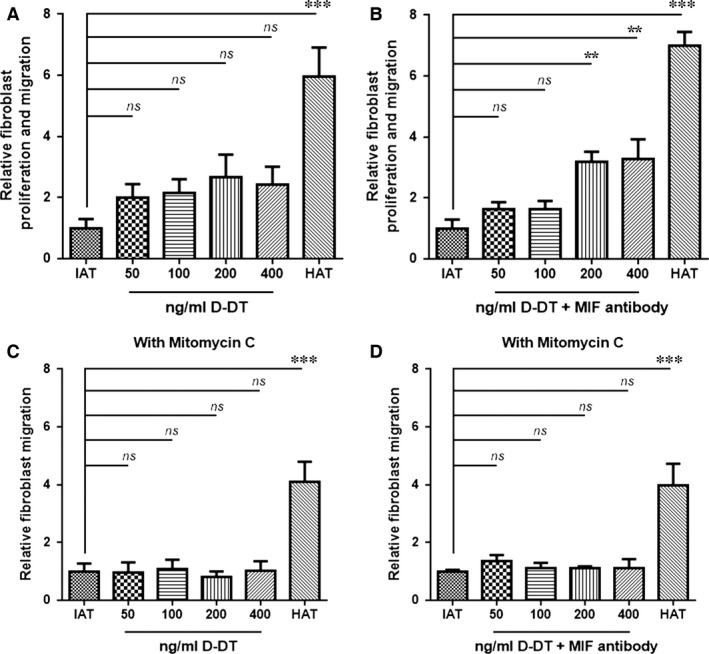
*In vitro* scratch assay of HDF and supernatants from IAT and HAT. Wound healing of HDF monolayers was evaluated by an *in vitro* scratch assay. Fibroblasts were incubated with supernatants of samples from IAT and HAT containing varying concentrations of recombinant human D‐DT and neutralizing MIF antibody. Medium containing 10% FCS served as a positive control and medium containing 0.5% FCS served as a negative control. Experiments were performed in the absence and the presence of the proliferation inhibitor Mitomycin C. (**A**) Supernatants from IAT showed no significant increase in proliferation and migration of fibroblasts when stimulated with D‐DT alone. (**B**) When D‐DT and anti‐MIF were added simultaneously, fibroblast proliferation and migration were increased significantly. (**C** and **D**) Fibroblast migration did not differ when Mitomycin C was added. All measurements were normalized to the negative control, which was set as 1. Data are presented in relative migration distance of fibroblasts ± S.E.M. *n* = 8–12 from 3 independent experiments, one‐way anova with Tukey's multiple comparisons test. Statistically significant differences are indicated by asterisks (**P < 0.01, ***P < 0.001).

We further used a standardized *in vitro* scratch assay that evaluates the proliferation and migration of HDF from the wound edges. Mitomycin C was optionally added to eliminate the proliferation of HDF and solely focus on the migration. Within the range of 50–200 ng/ml recombinant D‐DT, a trend towards increased wound healing was detected, but a statistically significant dose‐dependent effect was not observed (Fig. 3A). When mitomycin C was added, the addition of D‐DT did not lead to an improved healing of HDF monolayers (Fig. 3C). However, when neutralizing anti‐MIF antibodies and recombinant D‐DT were added simultaneously, the proliferation of HDF increased significantly (Fig. 3B). These results suggest that D‐DT may have an opposing effect on wound repair which becomes apparent when MIF is blocked, and that the process is primarily based on HDF proliferation rather than migration (Fig. 3D).

### Differential expression of MIF and D‐DT receptors in human and murine adipose tissue

**Figure 4:**
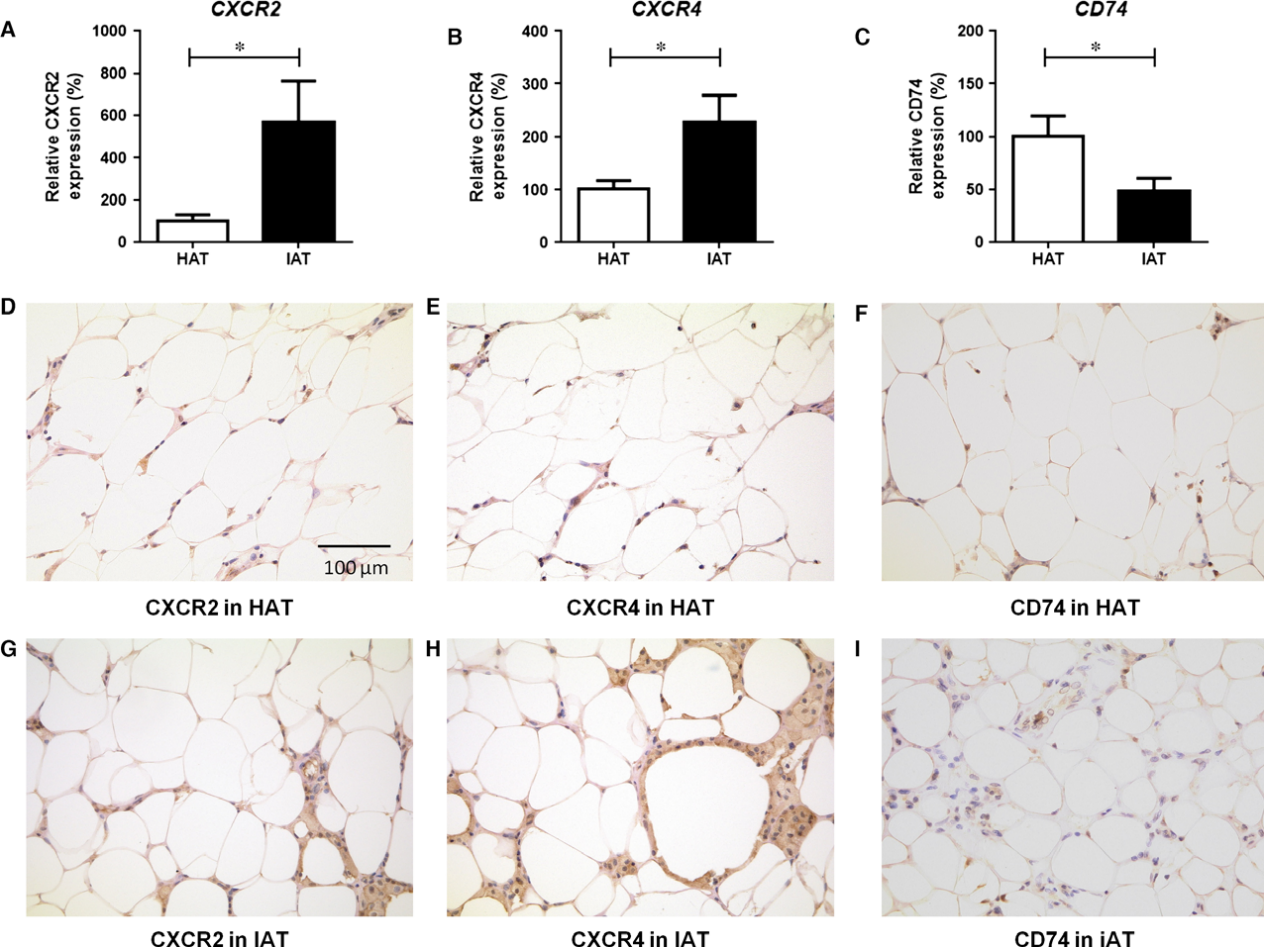
Expression of the receptors CXCR2, CXCR4, and CD74 in human HAT and IAT samples. (**A**–**C**) RNA was isolated from HAT and IAT, Gene expression for (**A**) *CXCR2*, (**B**) *CXCR4* and (**C**) *CD74* were determined and gene expression was analysed to *GAPDH*. *CXCR2* and *CXCR4* expression was increased in human samples from IAT samples. The mRNA levels for *CD74* showed a decrease in IAT samples. Data are presented as mean ± S.E.M., *n* = 12–16, two‐tailed Student's *t*‐test. Statistically significant differences are indicated by asterisks (*P < 0.05). (**D**–**I**) Additionally, immunohistological staining of HAT and IAT samples was performed.

**Figure 5:**
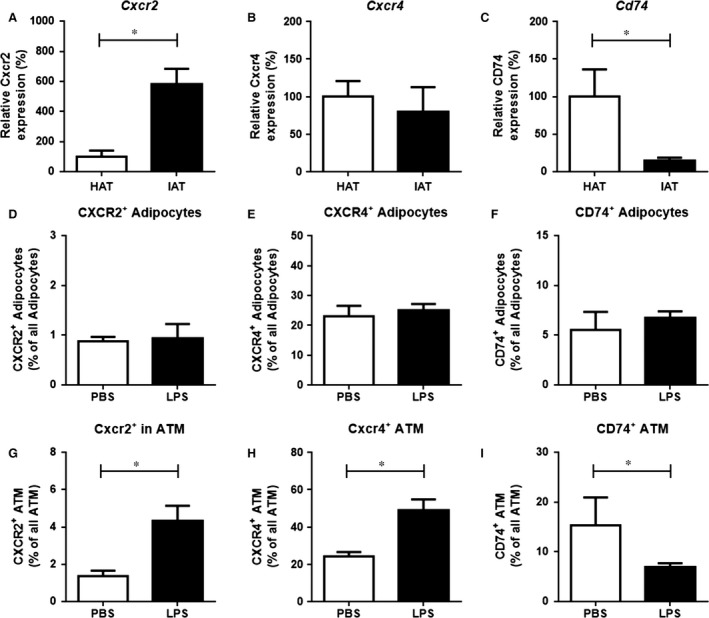
Expression of the receptors CXCR2, CXCR4, and CD74 in murine epididymal adipose tissue after PBS and LPS challenge. (**A**–**C**) RNA was isolated from LPS challenged murine epididymal fat pads. PBS injected murine epididymal fat pads were used as control. Gene expression for (**A**) *Cxcr2*, (**B**) *Cxcr4* and (**C**) *Cd74* were determined and gene expression was analysed to β*‐actin*. After LPS challenge in epididymal fat tissue, *Cxcr2* mRNA levels were increased in the adipose tissue, while *Cxcr4* expression was unaltered. *Cd74* expression was decreased after LPS injection. (**D**–**I**) Recepter expression on adipocytes and ATM was studied by flow cytometry. (**D**–**F**) In adipocytes, receptor expression did not differ significantly. (**G**–**I**) In ATM, by contrasts, CXCR2 and CXCR4 were significantly up‐regulated in LPS injected fat pads, whereas CD74 was down‐regulated. Data are presented as mean ± S.E.M., *n* = 5–5, two‐tailed Student's *t*‐test. Statistically significant differences are indicated by asterisks (*P < 0.05).

MIF and D‐DT both bind and signal through the CD74/CD44 receptor complex 15. The chemokine receptors CXCR2 and CXCR4 also have been described as receptors for MIF 24. The mRNA expression of *CXCR2* and *CXCR4* was up‐regulated in human IAT (Fig. 4A and B) and in mice a similar up‐regulation in gene expression after LPS injection was observed for *Cxcr2* in epididymal fat pads (Fig. 5A). However, the mRNA levels for *Cxcr4* were not altered (Fig. 5B) in mouse adipose tissue. Interestingly, expression of *CD74* was down‐regulated in both human IAT (Fig. 4C) as well as in mouse adipose tissue (Fig. 5C). Immunohistochemical staining of CXCR2, CXCR4 and CD74 confirmed the results of the qPCR analysis and indicated an up‐regulation of CXCR2 and CXCR4 in IAT, whereas CD74 was down‐regulated (Fig. 4D–I). Finally, flow cytometry analysis of murine adipose tissue revealed that the increase in CXCR2 and CXCR4, and the decrease in CD74 primarily occurred in macrophages, whereas receptor expression in adipocytes did not differ (Fig. 5D–I).

### Decreased D‐DT levels in mouse adipose tissue after LPS stimulation

Mouse D‐DT levels were measured in the epididymal fat pads one day after injection of LPS into epididymal fat pads. D‐DT levels decreased in the LPS challenged group when compared to the PBS‐injected control group (Fig. 5A), recapitulating our observations in human HAT and IAT.

### D‐DT exhibits decreased macrophage recruitment into mouse adipose tissue after LPS injection

**Figure 6:**
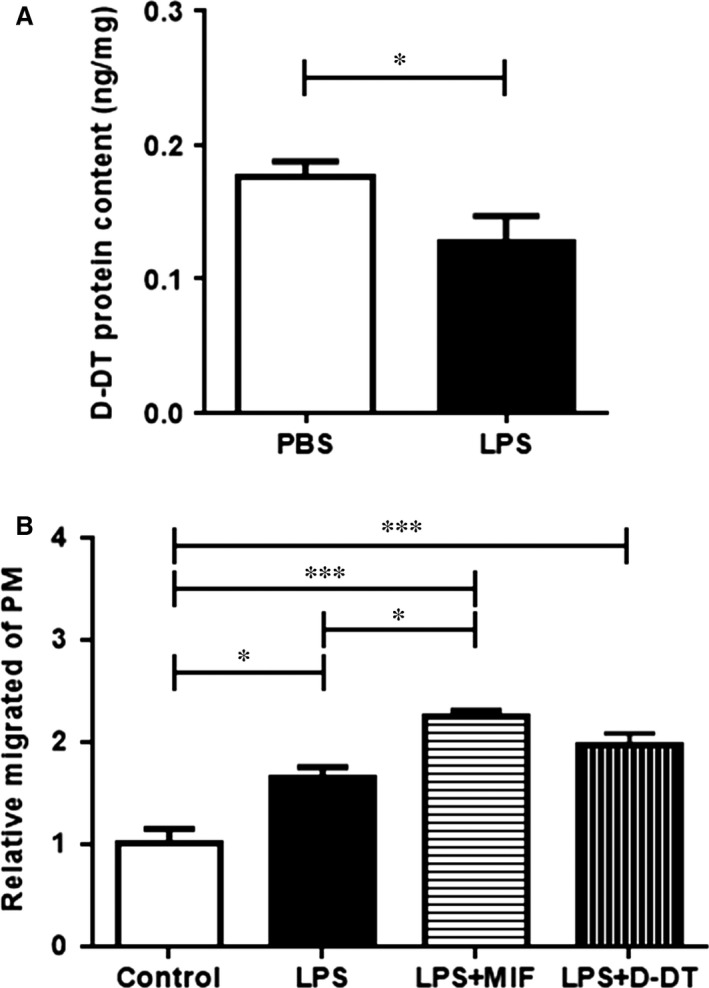
D‐DT levels in LPS challenged mouse epididymal adipose tissue and *in vivo* cell tracking of labelled peritoneal macrophages (PM). (**A**) Murine epididymal fat pads were challenged by LPS injection and the D‐DT levels were measured by ELISA. D‐DT levels in LPS challenged epididymal fat pads were significantly diminished compared to the PBS injected control group. Data are presented as mean ± S.E.M., *n* = 6–7, two‐tailed Student's *t*‐test. (**B**) FITC‐labelled peritoneal macrophages were injected retro‐orbitally into WT mice after inducing a local inflammation in the epididiymal fat pads by LPS. Additionally, the fat pads were also injected with recombinant MIF, D‐DT and PBS as control. After 48 hrs, mice were killed and FITC‐positive macrophages were measured by flow cytometry. Doublets were excluded by sideward scatter (SSC) and forward scatter (FSC), macrophages were defined as CD45, CD11b, and F4/80 positive cells. LPS injection led to an increase of migrated PM when compared to PBS. The results also showed that injection of LPS and MIF led to a higher PM accumulation in epididymal fat pads compared to the injection of LPS only, whereas no such effect was seen for LPS and D‐DT. Data are presented as % of FITC‐positive PMs ± S.E.M. *n* = 4–5 samples, from two independent experiments, one‐way anova with Dunnett's multiple comparisons test. Statistically significant differences are indicated by asterisks (*P < 0.05, ***P < 0.001).

To further elucidate the functional role of D‐DT in IAT, we performed an *in vivo* cell tracking experiment. Labelled PMs were injected retro‐orbitally into mice after induction of a local inflammatory response in epididiymal fat pads by a combination injection of LPS/MIF/D‐DT. When compared to the control group (PBS injection), adipose tissue from LPS treated mice showed increased PM invasion. Injection of LPS and MIF led to an increased PM accumulation in the epididymal fat pads that was significantly higher than in the LPS treated group. An increase in PM content was also observed when D‐DT was co‐injected with LPS; however, this effect was lower compared to MIF and did not reach statistical significance (Fig. 6B).

## Discussion

In this study, we observed a decrease in D‐DT expression at both the protein and mRNA levels in IAT when compared to HAT. The first study to describe D‐DT as an adipokine was provided by Iwata *et al*., who showed that D‐DT was mainly expressed in adipocytes and not SVF cells 11. Furthermore, the study showed that *D‐DT* mRNA was expressed during the course of adipogenic differentiation only in differentiated adipocytes. Interestingly, in this study the mRNA expression of *D‐DT* in adipocytes correlated negatively with the donor's BMI. Our study could not confirm a negative correlation of D‐DT levels with donor's BMI; however, this may be attributed to the fact that our study focused on the D‐DT levels in entire subcutaneous adipose tissue rather than only adipocyte‐specific D‐DT. These observations stand in contrast to MIF, which shows a positive correlation with BMI and obesity, and has been shown to promote adipose tissue inflammation 16, 25, 26, 27. In our study, immunohistochemical staining revealed that in healthy tissue D‐DT expression was overall diminished in the adipocytes of IAT when compared to controls. A recent study showed that MIF is expressed in both adipocytes as well as SVF cells and that within inflamed adipose tissue MIF mRNA expression and protein levels were significantly increased 4. These findings point to potentially opposite functions of D‐DT and MIF in adipose tissue and wound repair.

Many studies have described wound healing to be impaired in obese patients 9. Favourable effects improving wound repair involve keratinocyte and fibroblast migration, proliferation, differentiation as well as keratinocyte and fibroblast interactions 28. We showed that supernatants from IAT had a harmful effect on the viability of HDF. But when co‐stimulated with D‐DT, the harmful effect of IAT supernatants was partially reversed and the cell viability and proliferation increased in a D‐DT dose‐dependent manner. These observations indicate that soluble factors from IAT have detrimental effects on HDF cell viability and proliferation and that the reduction of D‐DT in IAT may play a role. D‐DT alone did not show significant effects on HDF in the scratch assay. The simultaneous addition of MIF antibody, which was shown to have beneficial effects on wound healing by impacting on fibroblast cell migration 4, and recombinant D‐DT, however, resulted in a significant increase in HDF proliferation further suggesting that MIF and D‐DT may have opposing effects to fibroblast wound repair. Although the exact mechanisms by which D‐DT exerts its effects are unknown, the activation of kinases such as the extracellular‐signal regulated kinases 1/2 may be a possibility 13.

Tissue resident macrophages can either arise from hematopoietic or from embryonic progenitor cells and they fulfill tissue‐specific and niche‐specific functions 29. Previous studies report that during obesity most adipose tissue resident macrophages originate from infiltrating blood monocytes and some studies have suggested that the migration and extension of macrophages in adipose tissue leads to increased local inflammation, which in turn could impact wound healing processes 30, 31, 32. MIF and D‐DT have been described to inhibit the chemotaxis of peripheral monocytes 13. When LPS was injected into epididymal fat pads, D‐DT protein levels were decreased, similar to decreased D‐DT levels in IAT. When murine epididymal fat pads were challenged with LPS plus MIF or D‐DT, only LPS plus MIF led to a significant increase in invaded PM when compared to the LPS treated group. Our data suggest that MIF may be the more dominant factor in macrophage recruitment than D‐DT.

Presently, little is known about the expression of MIF/D‐DT receptors in adipose tissue and their association with adipose tissue inflammation. Both MIF as well as D‐DT bind to the cell surface receptor CD74, but only MIF has been described to signal through the non‐cognate receptors CXCR2 and CXCR4 15. Our study showed an increased expression of CXCR2 and CXCR4 in IAT, elevated levels of CXCR2 in challenged mouse adipose tissue, while the expression of CD74 was down‐regulated in adipose tissue inflammation in both humans and mice. Both CXCR2‐mediated signalling, as well as CXCR4‐mediated signalling have been implicated with adipose macrophage and monocyte recruitment and the CD74/CD44 complex has been shown to promote cell survival and pro‐inflammatory responses 15, 24, 33. MIF expression was shown to be increased in human IAT, and promoted macrophage infiltration to site of inflammation 4, correlating with our data of increased *CXCR2/CXCR4* expression in human tissue. By flow cytometry we also showed that the up‐regulation of CXCR2 and CXCR4 is mainly found on macrophages, whereas receptor expression on adipocytes was unaltered. Recent studies have described the involvement of macrophage recruitment in acutely inflamed adipose tissue, which occurred in combination with decreased expression of intercellular adhesion molecule‐1 and CD44, two relevant adhesion molecules inducing monocyte infiltration 4, 27. As CD74 needs CD44 for its beneficial functions (*e.g*. cell proliferation), the observed decrease of CD44 expression is an indication that CD74 also is not involved in this context; this correlates well with the decrease in CD74 expression found in our study. Together with the *in vivo* experiments, our results suggest that macrophage recruitment into IAT is primarily promoted by MIF and CXCR2/4 rather than through the D‐DT‐CD74 axis.

We have to acknowledge some limitations of the present work which is an initial study to examine a role for D‐DT during acute adipose tissue inflammation and wound repair. First, the studies examining the effect of D‐DT on HDF were performed in an *in vitro* setting in an artificial environment and have to be interpreted with caution. While the tissue expression of D‐DT is low, high concentrations of recombinant D‐DT were necessary to see effects in the *in vitro* viability, proliferation and wound healing assays. Definite conclusions cannot be made until additional *in vivo* studies are performed. Second, the LPS injection model used in our *in vivo* experiment is a simplified model that does not mimic the more complex wound healing disorders of the human samples. We also acknowledge that LPS was injected into epididymal fat pads and not into subcutaneous adipose tissue which may be a more appropriate tissue type.

Clearly, it would be interesting to further investigate the specific function of D‐DT in wound repair in more extended studies, for example, using an *in vivo* wound model, preferably including an adipose tissue conditional knockout of *D‐dt*. Furthermore, in our study we only have focused on whole adipose tissue without differentiation of the respective cell fractions. Therefore, it may be interesting to investigate changes in MIF, D‐DT, and receptor expression after separation into adipose tissue (*e.g*. after collagenase digestion) in future studies.

**Figure 7:**
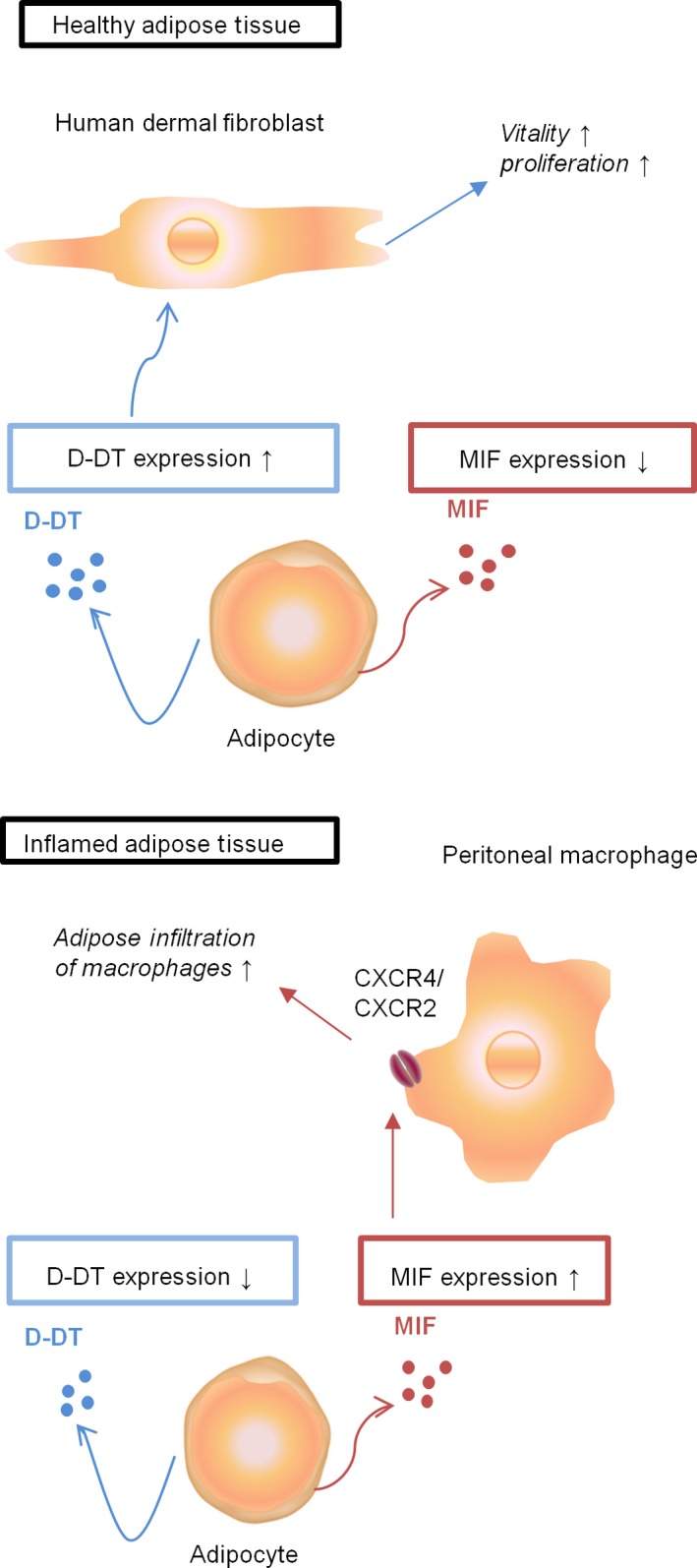
Schematic illustration of the function of D‐DT and MIF in adipose tissue. Our data suggest that within healthy adipose tissue, D‐DT may increase the proliferation and vitality of human dermal fibroblasts. Upon inflammation the levels of D‐DT decrease, while MIF expression is enhanced, which subsequently may lead to an increase in peritoneal macrophage infiltration through MIF/CXCR4 or MIF/CXCR2 signalling, suggesting a pathological role for MIF in adipose tissue.

In conclusion, our study indicates that D‐DT may play a distinct role from MIF in adipose tissue inflammation during wound healing disorders. In contrast to MIF, D‐DT protein and mRNA levels were decreased in IAT, which may be detrimental for wound healing since supplementation with recombinant D‐DT led to significantly increased HDF viability and proliferation. Furthermore, our data suggest that in contrast to D‐DT, MIF is the more potent chemokine that facilitates the recruitment of macrophages to the inflammatory site by its receptors CXCR2/4. A schematic illustration of our findings and the reciprocal roles of MIF and D‐DT in adipose tissue is demonstrated in Figure 7. However, additional *in vivo* studies are required especially to draw a final conclusion regarding the effect of D‐DT on fibroblasts as the *in vitro* studies show several major limitations.

## Conflict of interest

The authors confirm that there are no conflicts of interest.

## Supporting information


**Figure S1** Positve and negative control for D‐DT staining.

**Figure S2** Regression analysis of D‐DT and BMI.

**Table S1** Primer list.

**Table S2** Antibody list for Flow cytometry.

**Table S3** Details of HAT.

**Table S4** Details of IAT.
