# Supplementary figures and images for: D‐dopachrome tautomerase in adipose tissue inflammation and wound repair
Source: J Cell Mol Med. 2016 Sep 7;21(1):35–45. doi: 10.1111/jcmm.12936 (PMC5192814; doi:10.1111/jcmm.12936)

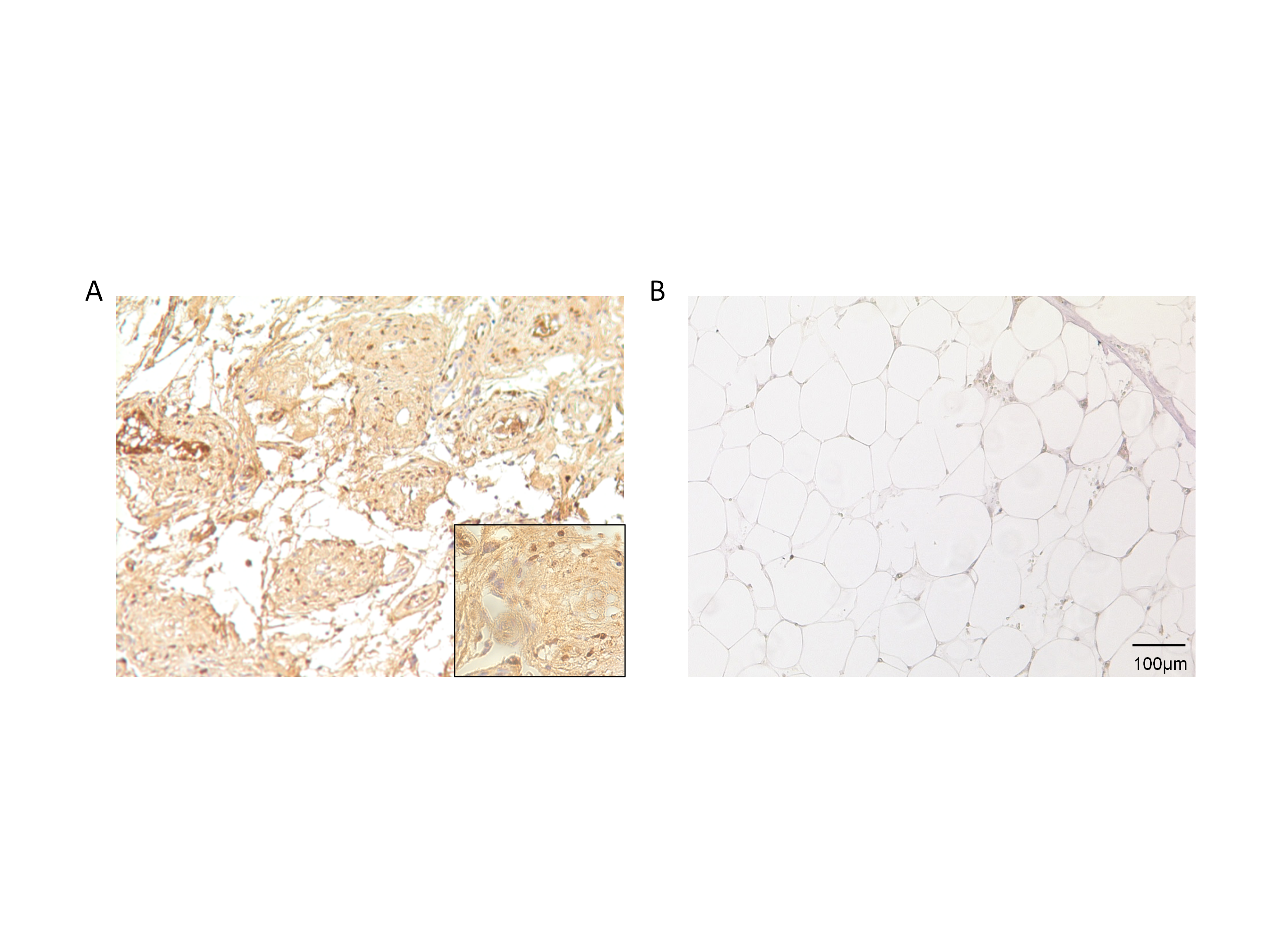

Supplement: Supplementary file 1 — Figure S1 Positve and negative control for D‐DT staining. [file JCMM-21-35-s001.tif]

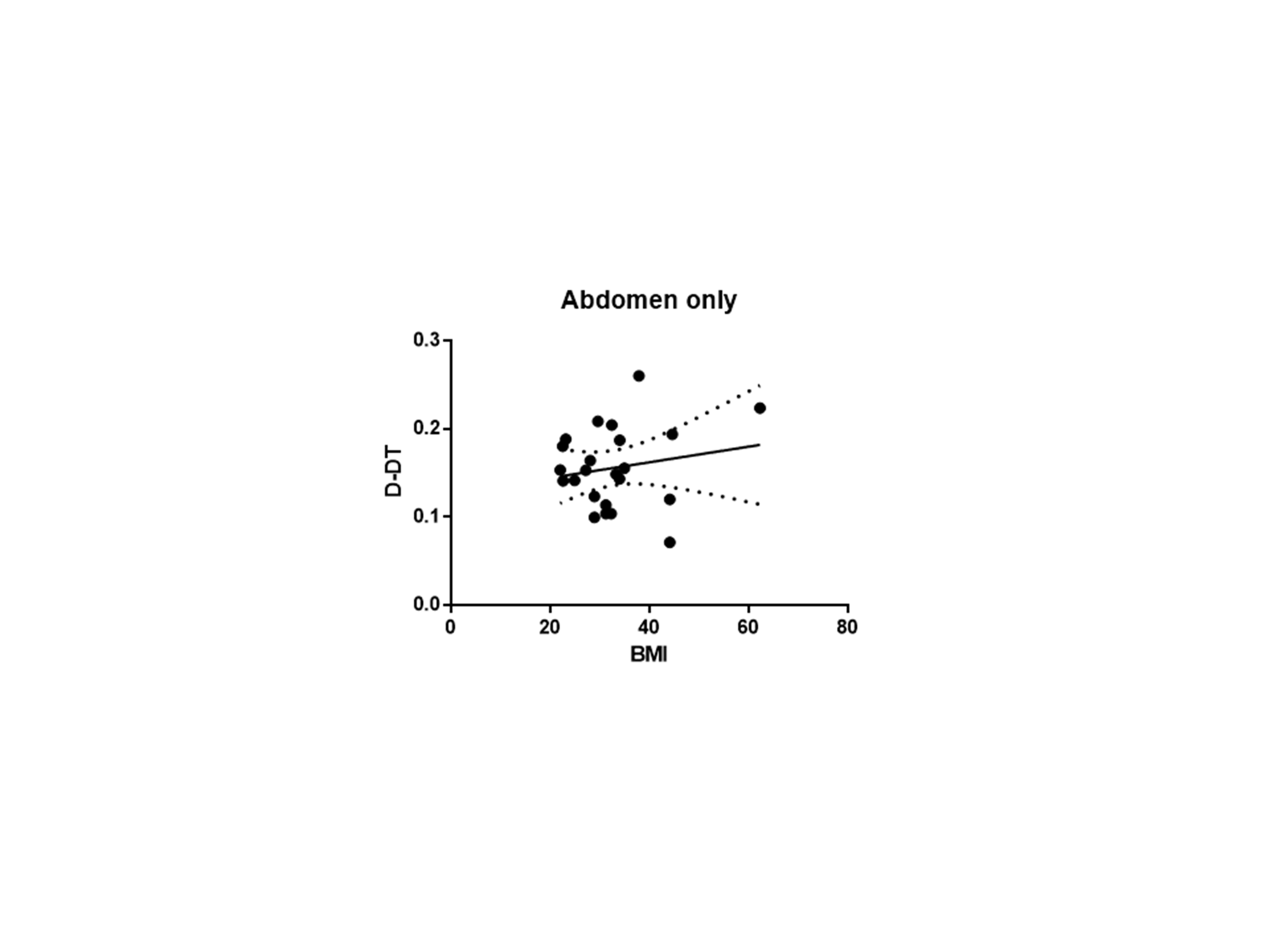

Supplement: Supplementary file 2 — Figure S2 Regression analysis of D‐DT and BMI. [file JCMM-21-35-s002.TIF]
