# Supplementary material for: D‐dopachrome tautomerase in adipose tissue inflammation and wound repair
Source: J Cell Mol Med. 2016 Sep 7;21(1):35–45. doi: 10.1111/jcmm.12936 (PMC5192814; doi:10.1111/jcmm.12936)
Supplement: Supplementary file 3 — Table S1 Primer list. [file JCMM-21-35-s003.doc]

| Primer | Species | Forward | Reverse |
| --- | --- | --- | --- |
| *GAPDH* | human | 5'-GAAGGTGAAGGTCGGAGTC-3' | 5'-GAAGATGGTGATGGGATTTC-3' |
| *D-DT* | human | 5'-CGCCCACTTCTTTGAGTTTC-3' | 5'-GGAAGAAGCAGCCAGTTCAC-3' |
| *CD74* | human | 5'-AGGTGACTGTCAGTTTGTCC-3' | 5'-TTTCCATCCTGGTGACTCTG-3' |
| *CXCR2* | human | 5'-CAATGAATGAATGAATGGCTAAG-3' | 5'-AAAGTTTTCAAGGTTCGTCCGTGTT-3' |
| *CXCR4* | human | 5'-ATCCCTGCCCTCCTGCTGACTATTC-3' | 5'-GAGGGCCTTGCGCTTCTGGTG-3' |
| *β-Akt* | mouse | 5'-GGCTGTATTCCCCTCCATCG-3' | 5'-CCAGTTGGTAACAATGCCATGT-3' |
| *Cd74* | mouse | 5'-CAACGCGACCTCATCT-3' | 5'-TGTTGCCGTACTTGGTAA-3' |
| *Cxcr2* | mouse | 5'-ATCTTCGCTGTCGTCCTTGT-3' | 5'-CACAGGTCTCCTTGATCA-3' |
| *Cxcr4* | mouse | 5'-TTCTCATCCTGGCCTTCATC-3' | 5'-CTTTTCAGCCAGCAGTTTCC-3' |

**Supplementary Table 1:** **Primer list**. Listed are primer sequences, which were used for quantification of mRNA levels in human and mouse tissue by real time-polymerase chain reaction.
