# Supplementary material for: D‐dopachrome tautomerase in adipose tissue inflammation and wound repair
Source: J Cell Mol Med. 2016 Sep 7;21(1):35–45. doi: 10.1111/jcmm.12936 (PMC5192814; doi:10.1111/jcmm.12936)
Supplement: Supplementary file 4 — Table S2 Antibody list for Flow cytometry. [file JCMM-21-35-s004.doc]

**Supplementary Table 2:** **Antibody list for Flow cytometry**. Listed are antibodies, which were used for flow cytometric staining of CXCR2, CXCR4, and CD74 in adipose tissue macrophages (ATM) and adipocytes from LPS and PBS injected mice.

| Target Cell | Antibody with Fluorophore | Manufacturer |
| --- | --- | --- |
| *ATM* | Cd11b-AlexaFluor700 | eBioscience, CA, USA |
| *ATM* | F4/80-eFluor450 | eBioscience, CA, USA |
| *ATM* | Cd45-PE | Biolegend, CA, USA |
| *ATM and adipocytes* | CXCR2-PerCP | BioLegend, CA, USA |
| *ATM and adipocytes* | CD74-FITC | BD Pharmingen, CA, USA |
| *ATM and adipocytes* | CXCR4-APC | eBioscience, CA, USA |
| *Adipocytes* | CR4-APC | eBioscience, CA, USA |
