# Supplementary material for: D‐dopachrome tautomerase in adipose tissue inflammation and wound repair
Source: J Cell Mol Med. 2016 Sep 7;21(1):35–45. doi: 10.1111/jcmm.12936 (PMC5192814; doi:10.1111/jcmm.12936)
Supplement: Supplementary file 5 — Table S3 Details of HAT. [file JCMM-21-35-s005.doc]

**Supplementary Table 3:** **Details of HAT**. Listed are information regarding age, gender and BMI of patients from which of healthy adipose tissue was collected.

| **Number** | **Gender** | **Age** | **BMI** |
| --- | --- | --- | --- |
| 1 | m | 33 | 30.67 |
| 2 | m | 54 | 25.83 |
| 3 | w | 27 | 24.22 |
| 4 | w | 56 | 29.39 |
| 5 | m | 24 | 37.88 |
| 6 | m | 22 | 25.31 |
| 7 | m | 35 | 33.95 |
| 8 | w | 38 | 25.73 |
| 9 | m | 65 | 27.17 |
| 10 | w | 71 | 18.67 |
| 11 | m | 16 | 25.74 |
| 12 | m | 57 | 29.41 |
| 13 | m | 36 | 25.37 |
| 14 | m | 51 | 28.91 |
| 15 | w | 53 | 31.62 |
| 16 | w | 81 | 20.57 |
| 17 | w | 45 | 29.64 |
| 18 | w | 80 | 33.25 |
| 19 | w | 80 | 33.25 |
| 20 | w | 54 | 44.10 |
| 21 | w | 54 | 44.10 |
| 22 | w | 31 | 35.43 |
| 23 | w | 39 | 31.22 |
| 24 | w | 54 | 44.62 |
| 25 | w | 48 | 27.24 |
| 26 | w | 54 | 25.34 |
| 27 | m | 51 | 28.91 |
| 28 | w | 21 | 62.28 |
| 29 | w | 29 | 27.34 |
| 30 | m | 27 | 32.27 |
| 31 | w | 52 | 32.41 |
| 32 | m | 61 | 33.25 |
| 33 | w | 53 | 31.22 |
| 34 | m | 44 | 22.79 |
| 35 | m | 26 | 19.38 |
| 36 | w | 48 | 27.94 |
| 37 | w | 73 | 22.04 |
| 38 | m | 51 | 34.94 |
| 39 | m | 30 | 34.02 |
| 40 | w | 45 | 23.14 |
| 41 | w | 44 | 22.59 |
| 42 | w | 47 | 22.49 |
| 43 | w | 54 | 47.63 |
| 44 | m | 62 | 33.08 |
| 45 | w | 47 | 24.91 |
| 46 | w | 61 | 33.98 |
| 47 | w | 43 | 37.18 |
| 48 | m | 28 | 27.14 |
| 49 | m | 59 | 27.17 |
| 50 | w | 41 | 21.46 |
| 51 | w | 17 | 28.06 |
| 52 | m | 38 | 29.07 |
| 53 | m | 57 | 25.25 |
| 54 | m | 52 | 21.13 |
