# Supplementary material for: D‐dopachrome tautomerase in adipose tissue inflammation and wound repair
Source: J Cell Mol Med. 2016 Sep 7;21(1):35–45. doi: 10.1111/jcmm.12936 (PMC5192814; doi:10.1111/jcmm.12936)
Supplement: Supplementary file 6 — Table S4 Details of IAT. [file JCMM-21-35-s006.doc]

**Supplementary Table 4:** **Details of IAT**. Listed are information regarding age, gender, BMI of patients and specification of the collected acutely inflamed adipose tissue.

| **Number** | **Gender** | **Age** | **BMI** | **Specification** |
| --- | --- | --- | --- | --- |
| 1 | m | 55 | 27.18 | Iatrogenic superficial thrombophlebitis |
| 2 | w | 64 | 21.80 | wound healing disorder after external trauma |
| 3 | m | 44 | 31.14 | Iatrogenic superficial thrombophlebitis |
| 4 | m | 76 | 35.43 | wound healing disorder after external trauma |
| 5 | w | 26 | 20.71 | wound healing disorder after external trauma |
| 6 | m | 25 | 21.01 | Iatrogenic superficial thrombophlebitis |
| 7 | m | 68 | 27.77 | Iatrogenic superficial thrombophlebitis |
| 8 | m | 42 | 19.58 | wound healing disorder after external trauma |
| 9 | m | 62 | 26.73 | wound healing disorder after external trauma |
| 10 | m | 83 | 26.79 | wound healing disorder after external trauma |
| 11 | w | 39 | 34.89 | postoperative wound healing disorder |
| 12 | w | 40 | 89.84 | postoperative wound healing disorder |
| 13 | w | 38 | 34.89 | wound healing disorder after external trauma |
| 14 | m | 40 | 32.10 | postoperative wound healing disorder |
| 15 | m | 71 | 46.24 | wound healing disorder after external trauma |
| 16 | w | 71 | 18.67 | wound healing disorder after external trauma |
| 17 | m | 75 | 52.24 | wound healing disorder after external trauma |
| 18 | w | 25 | 23.44 | postoperative wound healing disorder |
| 19 | m | 69 | 27.77 | postoperative wound healing disorder |
| 20 | m | 70 | 34.02 | postoperative wound healing disorder |
| 21 | w | 70 | 26.03 | wound healing disorder after external trauma |
| 22 | w | 52 | 25.01 | wound healing disorder after external trauma |
| 23 | m | 40 | 32.10 | postoperative wound healing disorder |
| 24 | w | 52 | 19.38 | wound healing disorder after external trauma |
| 25 | w | 53 | 25.39 | wound healing disorder after external trauma |
| 26 | w | 29 | 27.34 | wound healing disorder after external trauma |
| 27 | w | 36 | 21.47 | wound healing disorder after external trauma |
| 28 | m | 62 | 33.83 | wound healing disorder after external trauma |
